# Consensus statement on best practices for refugee care in Wisconsin

**DOI:** 10.1186/s12919-017-0072-y

**Published:** 2017-06-13

**Authors:** Jim Sanders, Hilary Chavez, S. Michele Cohen, Mary Enright, Mary Flynn, Tifany Frazer, Kelly Hoormann, Ben Rader, Sebastian Ssempijja, Samantha L. Wilson

**Affiliations:** 1 Department of Family and Community Medicine, Medical College of Wisconsin, Milwaukee, WI USA; 2 Medical College of Wisconsin, Milwaukee, WI USA; 3 Choice Consulting Services, LLC, Milwaukee, WI USA; 4 Sebastian Family Psychology Practice, Milwaukee, WI USA; 5 Lutheran Social Services of Wisconsin and Upper Michigan, Milwaukee, WI USA; 6 Office of Global Health, Medical College of Wisconsin, Milwaukee, WI USA; 7 Institute for Health and Society, Medical College of Wisconsin, Milwaukee, WI USA; 8 Department of Pediatrics, Medical College of Wisconsin, Milwaukee, WI USA

**Keywords:** Refugee, Immigrant health, Health services, Inter-professional collaboration

## Background

The State of Wisconsin has a long history of refugee resettlement with over 70,000 refugees having come to Wisconsin from all over the world. At present, Wisconsin welcomes around 1500 refugees every year with the majority being resettled in the state’s urbanized southeastern corridor. Refugee resettlement trends within Wisconsin have fluctuated in conjunction with worldwide political changes. In the early to mid-1980’s, after the fall of Laos, there was a vast influx of Southeast Asian migration to Wisconsin. Following the dissolution of the former Soviet Union (marked with the destruction of the Berlin Wall in 1989), refugees from Eastern Europe (and the former regions within USSR) entered Wisconsin. Subsequently, people with Slavic and Illyrian ancestry immigrated to Wisconsin as refugees after the collapse of the Former Republic of Yugoslavia. This last large migration peaked towards the end of 1999, a time when it was typical for Wisconsin to receive refugees from only a handful of countries every year. In fact, up until the late 1990’s well over 70% of Wisconsin’s annual refugee resettlement originated from just three regions: Southeast Asia, the former Soviet Union and the former Yugoslavia Republic.

But after the turn of the century, trends in Wisconsin’s refugee resettlement began to show much more diversification in country of origin. By 2003, for example, the number of countries of origin represented in Wisconsin’s refugee arrivals grew to fifteen; 70% of whom were arriving from sub-Sahara Africa. This dramatic increase in countries of origin also meant that the religions, cultures, language groups and skill sets represented within Wisconsin’s arriving refugees also became more varied. This trend towards increased diversity within the refugee population entering Wisconsin has held fairly consistent over the last 15 years. Continued worldwide conflicts and ongoing, entrenched political and cultural tensions would imply that this trend represents a new normal in refugee resettlement. That is, while the number of distinct countries might be relatively stable, the names of the countries shifts from year to year. In 2015, for example, resettled refugees arrived from 17 countries representing South Asia, Central Asia, Middle East, Caribbean Basin, Sub-Saharan Africa, North Africa and Southeast Asia.

The local response to resettling such a widely diverse refugee population is challenging. Providing culturally attuned healthcare, social and governmental services to assist with the relocation and acculturation of such a divergent collection of individuals is a continuous task. Support services must often work quickly to properly introduce and orient recently resettled refugees into their new communities and promote emerging autonomy within the host culture. The task of helping refugees adjust to Wisconsin is complex: acquiring housing, coordinating health services, English language education, school enrollment, job placement and more. Wisconsin has a good track record of serving the needs of its refugee population with 95% (66,500) of all the refugees who have ever settled in Wisconsin have achieved economic self-sufficiency, are working and contributing to their communities, and almost all are now U.S. citizens [1].

However, this success has not come without significant challenges largely born by the many individuals and agencies who provide culturally sensitive and empathetic services to refugees and new immigrants. Over the last few years anecdotal remarks have been picking up in both frequency and urgency that the work to resettle refugees successfully has become more difficult and that resources are having to be stretched thinner and thinner. Some of this stress has been aggravated by the general public’s perception that refugees pose a threat to their own safety and security and there is mounting anti-immigrant sentiment.

Surprisingly, and despite sharing similar goals and clients, programs and services aimed at supporting refugee populations are often disconnected from one another. As unique competencies are achieved with each new refugee group, the work starts anew as another sub-population arrives; resulting in an endless tide of diverse challenges for providers (language, culture, trauma, etc.). And more and more often, multiple diverse populations are presenting various challenges at the same time which further increases the disconnection due to time requirements and competing demands.

It was against this backdrop of rapidly shifting cultural/language needs of resettled refugees, shrinking governmental budgets and increased public sentiment against refugee immigration that an inter-agency, inter-disciplinary group of professionals came together in December of 2014 in order to better understand the ways refugee services might be more thoughtfully delivered. This “Working Group” met over several months and varied in both size and make-up across meetings, but included a core group of 6–8 people representing the fields of anthropology, medicine, nursing, physical therapy, psychology, public health, nursing and social work.

After several meetings to establish trust, a shared vision and identify collective resources the Working Group recognized that the shifting landscape of refugee resettlement meant that no-one central agency or entity was all-knowing; expertise resided across numerous people and agencies (and at times with limited overlap). Therefore a multi-day conference was proposed with a purpose to “mobilize and harness the diverse knowledge, capacities and services of various stakeholders, interested to learn from and with those who interface with refugee and immigrant communities in the United States of America”.

## Action

One year later, the multi-disciplinary conference, entitled “Our City of Nations”, was held at the Medical College of Wisconsin (Milwaukee, Wisconsin) on December 3^rd^ and 4^th^, 2015. The first day focused on refugee health and wellness and the second day centered on immigrant health and wellness.

Over 200 participants from 62 agencies attended; participants represented 16 professional fields and 10 different regional cities. Concurrently, 127 graduate and undergraduate students from 11 academic institutions in the Milwaukee Metro-area (representing eight diverse degree programs) participated within an inter-professional educational refugee health session. Additionally, a photography exhibition entitled “Here, There and Elsewhere: Refugee Families in Milwaukee” provided a window into the daily lives of some of the newest, least familiar Wisconsin residents through a collection of 30 photographs taken by a well-known Milwaukee artist.

The conference was intentionally designed to be a networking forum for Wisconsin’s multidisciplinary professionals so that both their lessons learned and their best practices might be readily shared. By relying upon each other and drawing from collective insights, the southeastern Wisconsin region could strengthen capacity to respond to diverse refugee communities.

The conference was divided into four distinct tracks: Social Services; Medical/Nursing; Mental Health; and Education. This decision was made to allow for inter- and intra-discipline sharing and paired collaboration.

The conference schedule allowed for daily multidisciplinary plenary presentations and multiple break-out sessions each with their own learning objective. The plenary presentations included federal policy makers, Wisconsin government officials, service organization leaders, healthcare providers and local activists. The break-out sessions were designed so that the ideas generated during the plenary could be filtered through the context of each participants’ experiences. Specifically, break-out sessions were facilitated by a moderator who sought to elicit discussion and generate ideas grounded in participants’ experiences. These ideas were captured by dedicated scribes seated within each break-out session; the resulting descriptions of the break out session discussions yielded rich statements centered on the topical themes of the sessions. A concluding session was held at the end of each day where these statements were reviewed, discussed and electronically voted upon by the conference participants.

Those statements which reached high levels of group acceptance (super majority consensus with at least 66% of voters agreeing on their validity) were collated together to form the *Consensus Statement on Best Practices for Refugee Care in Wisconsin* (Additional file 1).

Additionally, participants’ feedback was documented through both paper evaluation forms and an online 10 question post-conference survey. Results indicate 1) that providers and professionals who attended believe that ongoing collaboration between all types of agencies, communities, navigators and government is necessary, 2) that private-public partnerships are essential in the ongoing efforts to adequately meet the health needs of recently arrived populations, and 3) significant enthusiasm for the conference, with the overwhelming majority attesting that attendance improved their ability to deliver services, provided relevant content and addressed themes meaningful to their work.

## Conclusions

This locally relevant conference began the building of a set of core competencies and integrative strategies to enhance the care for the State’s diverse refugee populations. The conference’s *Consensus Statement on Best Practices in Refugee Health in Wisconsin* provides a framework by which to approach refugee care services and to hold each other accountable for services delivered outside of our individual disciplines.

Milwaukee is by no means unique in many of the trends or challenges surrounding the refugee resettlement process. Other areas of the United States and world at large face similar struggles. The methods by which our community came together might be beneficial to these other refugee resettlement groups. The Consensus Statement might be used as a template by which other municipalities measure their own efforts and is readily adaptable to local situations.

By coming together and sharing our experiences with each other we can do better as individual providers, agencies and as a community.

## Additional file

Additional file 1: Consensus statement on best practices for refugee care in southeast Wisconsin. (DOCX 17 kb)
