# Supplementary material for: Consensus statement on best practices for refugee care in Wisconsin
Source: BMC Proc. 2017 Jun 13;11(Suppl 5):5. doi: 10.1186/s12919-017-0072-y (PMC5496018; doi:10.1186/s12919-017-0072-y)
Supplement: Additional file 1: — Consensus statement on best practices for refugee care in southeast Wisconsin. (DOCX 17 kb) [file 12919_2017_72_MOESM1_ESM.docx]

**Consensus statement on best practices for refugee care in southeast Wisconsin**

- Refugee resettlement requires a private-public partnership in an ongoing process involving community engagement.
- To meet the needs of the increasing number of new refugees, agencies need to work smarter and faster in a collaborative fashion to integrate the best possible experience of these refugees.
- In working with refugees, recognize individual differences (i.e. that each refugee is unique even within his/her culture) but remain mindful of customs and err towards modesty and simplicity with self-expression.
- The stated goal of resettlement is self-sufficiency. It’s not just safety or hospitality; it’s working with refugees so they are able to become self-sufficient citizens of our country and achieve their hopes and dreams.
- The refugee experience often includes circumstances that disrupt their cultural norms and reduce social support. Collectively, these may cause a loss of identity and distrust in government or larger systems.
- Be aware of your own narrative (cultural background and personal experiences) in your encounters with refugees, and be prepared to listen to refugees’ narratives at their own pace.​
- The social context of interpretation is multi-faceted; as such, consider the potential impact of *who* provides interpretive services. Avoid dual relationships, and consider the client’s comfort level with the interpreter.
- Different cultures may conceptualize pain, illness, and disease differently from “western-based” frameworks; one size does not fit all. We must find critical elements that translate when the usual words and Western definitions fail.​
- Providers must seek to maintain cultural humility when assessing individual patients. Providers must strive to know something about: history and culture, social structure, basic health and illness concepts, the spiritual aspects of health and illness, and key Western and cultural psychiatric/psychological concepts BEFORE asking questions.​
- To move towards health equity, we need to implement health in all policies with health equity as an explicit goal.
- Healthcare needs to change its focal point to helping communities better utilize primary care teams and community health workers so as to reduce reliance on emergency medicine.
- We need to keep communication between the government and communities strong so that priorities set at a community level can be reflected in government policies and budgets.
- More community navigators are needed to build relationships with community members, articulate and anticipate obstacles, and advocate for communities.
- Many refugees are eager to seek health services and to learn to navigate the health care system; we must work to address the challenges for both providers and refugees to help achieve this medical self-sufficiency.
